# Polypharmacy in older patients with diabetes mellitus: a population based-study of northern Italy

**DOI:** 10.1007/s00592-025-02523-1

**Published:** 2025-06-05

**Authors:** Elena Succurro, Luisa Ojeda-Fernández, Carlotta Franchi, Anna Zanovello, Laura Pierini, Alessandro Nobili, Ida Fortino, Giorgio Sesti, Marta Baviera

**Affiliations:** 1 Department of Medical and Surgical Sciences, University Magna Graecia of Catanzaro, Viale Europa, Catanzaro, 88100 Italy; 2 Research Center for the Prevention and Treatment of Metabolic Diseases (CR METDIS), University Magna Graecia of Catanzaro, Catanzaro, Italy; 3 Department of Health Policy, Istituto di Ricerche Farmacologiche Mario Negri IRCCS, Milan, Italy; 4 Unità Organizzativa Osservatorio Epidemiologico Regionale, Lombardy Region, Milan, Italy; 5 Department of Clinical and Molecular Medicine, Sapienza University of Rome, Rome, Italy

**Keywords:** Polypharmacy, Diabetes mellitus, Older patients, Antihyperglycemic drugs, Multimorbidity

## Abstract

**Aims:**

To evaluate the trends in chronic polypharmacy and identify predictors of polypharmacy exposure in a population residing in Lombardy region of Italy.

**Methods:**

Using an administrative health database, we identified individuals aged 65–90 years with diabetes mellitus (DM) treated with antihyperglycemic drugs from 2010 to 2022. The trend of chronic polypharmacy was assessed using the Cochran-Armitage trend test. An adjusted logistic regression model was employed to analyze predictors of polypharmacy exposure.

**Results:**

The number of older patients with DM increased from 243,160 in 2010 to 314,238 in 2022. The prevalence of polypharmacy exposure rose from 13.8% in 2010 to 15.8% in 2013, followed by a decline starting in 2014. Notably, in 2020, the prevalence dropped to 11.8%, further decreasing to 9.1% in 2021, before rising again to 11.7% in 2022. We also observed an increased use of recommended antihyperglycemic drugs over time. Significant predictors of polypharmacy exposure included advanced age, female sex, comorbidities, and use of DPP-4i, GLP-1-RA, insulin, and SGLT2-i.

**Conclusions:**

The observed decrease in polypharmacy in the latter years of the study period may reflect improvements in the management of older patients with DM, aligning with recommended therapies, particularly for those at higher risk of polypharmacy.

**Supplementary Information:**

The online version contains supplementary material available at 10.1007/s00592-025-02523-1.

## Introduction

Type 2 diabetes (T2D) is a common condition in the aging population [1]. It is estimated that more than 25% of people aged 65 years or older worldwide have T2D [1–3]. Older adults with diabetes have higher rates of coexisting illnesses, such as hypertension, coronary heart disease, and stroke, compared to those without diabetes [3]. Furthermore, older adults with T2D are at greater risk than their nondiabetic peers for several common geriatric syndrome that can lead to a significant increase in drug use [3, 4].

Notably, polypharmacy is a widespread condition among elderly people with diabetes, affecting over 60% of this population [5–8]. Polypharmacy is defined as the use of five or more medications also prescribed chronically, which is common among individuals with multimorbidity, as one or more medications may be prescribed to treat each condition [9]. It is associated with non-adherence, inappropriate prescriptions, and adverse outcomes, including mortality, falls, drug-drug interactions, adverse drug reactions, and hospitalization [5, 9–11]. In people with T2D, polypharmacy is linked to suboptimal glycemic control and an increased risk of hypoglycemia [5, 10].

On the other hand, polypharmacy is a legitimate response to multimorbidity and is often necessary for individuals with T2D and comorbidities, making it clinically appropriate. Notably, in people with T2D and cardiovascular disease (CVD), a combination of glucagon-like peptide-1 receptor agonists (GLP-1 RA), sodium-glucose cotransporter-2 inhibitors (SGLT2-i), antithrombotic, antihypertensive, and lipid-lowering medications has been shown to improve major CV adverse events (MACE) [8, 12–26]. Despite elderly patients, particularly the very old, are not sufficiently represented in trials, some meta-analyses have shown that in older patients, treatment with GLP-1 RA reduced the risk for MACE, consistent with their effect in the overall population. Additionally, treatment with SGLT-2i improved CV and renal outcomes, with a significant reduction in heart failure (HF) hospitalizations, similar to that observed in younger people with T2D [13–26]. Based on several evidence the American Diabetes Association and the European Association for the Study of Diabetes guidelines (GLs) strongly recommend the use of GLP-1 RA and SGLT2-i in patients with T2D at high CV risk or with established atherosclerotic CVD [27, 28] as well as the prescriptions of antihypertensive, antithrombotic, and lipid-lowering treatments for CV protection [3, 27, 28].

However, the overall impact of increased drug prescribing needs to be assessed and monitored, in relation to patient characteristics and the risk-benefit ratio of polypharmacy, and this is also an important issue in terms of health care costs.

In this study, we assessed the trend of chronic polypharmacy from 2010 to 2022, and the trend of antihyperglycemic drugs in a large cohort of older patients with diabetes mellitus (DM) residents in Lombardy, the most populous region of Italy. Additionally, we evaluated the prevalence of most prescribed drug classes and the predictors associated with exposure to polypharmacy in this population.

## Materials and methods

### Source data

In this study, the analysis was conducted using the administrative healthcare database of Lombardy region [29], the largest region in northern Italy, with a population of about 10 million residents, accounting for nearly 16% of Italy’s total population. The database includes demographic data, drug prescriptions, and hospital records from 2000 to 2022. A unique personal code identifies each individual, enabling the linkage of all datasets. To ensure privacy, each identification code is automatically de-identified before the dataset is provided to researchers. The inverse process is only permitted to the Regional Health Authority, and only upon request from judicial authorities. Access to the Lombardy healthcare administrative database was granted in accordance with an agreement between the Istituto di Ricerche Farmacologiche Mario Negri IRCCS and the Lombardy Welfare Directorate.

One of the datasets used is the pharmacy prescription database, which contains information on medication names, Anatomic Therapeutic Chemical (ATC) classification codes, quantities dispensed, and dispensing dates for drugs reimbursed by the National Health System (NHS). Another dataset is the hospital database, which includes information on patient admissions, discharges, deaths, primary diagnoses, and up to five co-existing clinical conditions and procedures. Diagnoses are uniformly coded according to the 9th International Classification of Diseases (ICD-9-CM) and are standardized across all Italian hospitals. These diagnoses are compiled by the hospital specialists directly responsible for patient care and are validated by hospitals against detailed clinical and instrumental data, as they determine reimbursement from the NHS.

In Italy, studies using aggregated, anonymous data from administrative databases that do not involve direct access by investigators to identifying information do not require Ethics Committee/IRB approval or notification, nor is patient informed consent necessary.

### Study population

The study population comprised older adults with DM, aged between 65 and 90 years, from 2010 to 2022. Subjects were selected based on at least two prescriptions of antihyperglycemic drugs within the same calendar year.

We excluded deceased individuals, those who had emigrated, and institutionalized patients for whom records of drugs dispensed by nursing homes were unavailable. The population was divided according to their exposure to chronic polypharmacy, defined as the prescription of five or more drugs during a one-month period for at least six months (whether consecutive or not), excluding antihyperglycemic drugs, in each considered year (index year). All drugs were classified according to the ATC system and grouped at the 4th level.

### Collected variables

The following patient characteristics were collected at baseline: age, sex, Health Protection Agencies (Agenzie di Tutela della Salute - ATS) of residence and history of hospitalization for predefined conditions of interest (cerebrovascular disease, ischemic heart disease, HF, atrial fibrillation (AF), peripheral artery disease, chronic kidney disease or end-stage kidney disease, liver disease, gastrointestinal disorders, chronic obstructive pulmonary disease and cancer) in the ten years prior to the index year. Additionally, prescriptions for medications of interest were recorded for the year before the index year. Hospitalizations and pharmacy prescriptions were classified according to the International Classification of Diseases, Ninth Revision (ICD-9), and the ATC classification code, respectively (Appendix).

### Statistical analysis

Descriptive analyses were performed to outline the baseline characteristics of the sample in 2010, 2016, and 2022 according to polypharmacy exposure. Categorical variables were expressed as frequencies and percentages, while continuous variables were reported as means ± standard deviations. The frequencies and percentages of prescriptions for antihyperglycemic drug classes were reported, stratified by polypharmacy exposure in 2010, 2016, and 2022. The presence of a statistically significant trend in polypharmacy prevalence from 2010 to 2022 was assessed using the Cochran-Armitage trend test. Polypharmacy prevalence was also calculated for each year, stratified by sex and age. A logistic regression model for chronic polypharmacy was fitted using baseline patient characteristics and index year as independent variables. Since SGLT-2i became available starting in 2015, an interaction term between this variable and the year was included in the model. Odds ratios (ORs) and their corresponding 95% confidence intervals (CIs) were estimated.

## Results

**Fig. 1:**
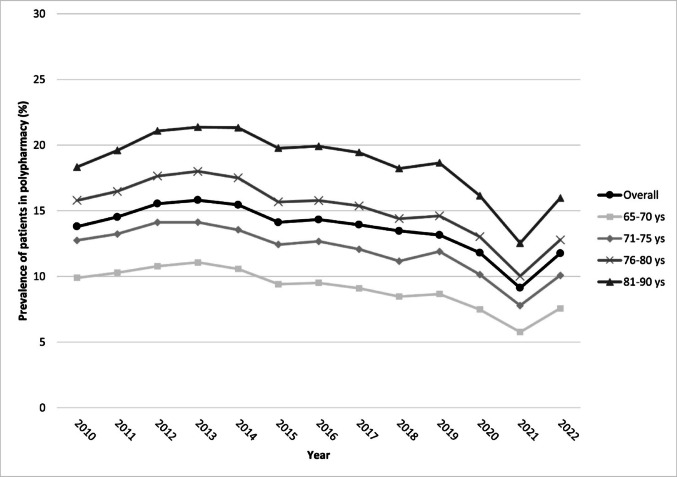
Trend of polypharmacy in older patients with diabetes, overall and stratified by age

The study population flowchart is shown in Fig. 1S. In our study population, the number of older patients with DM receiving prescriptions for antihyperglycemic drugs was 243,160 in 2010 and 314,238 in 2022, respectively. Table 1 depicts the baseline characteristics of older patients with DM according to polypharmacy in three years (2010, 2016, and 2022). Patients exposed to polypharmacy were slightly older, had more comorbidities, and received more medications compared to their counterparts in the three selected years. Overall, we observed a reduction in the percentages of patients with pre-existing clinical conditions, defined as hospitalization in the previous 10 years, from 2010 to 2022 in both the polypharmacy and control groups. Furthermore, a higher prevalence of hospitalization for at least one comorbidity was reported in patients in the polypharmacy group compared to those in the non-polypharmacy group, although a trend toward a decrease was observed in both groups over time. These changes were accompanied by a reduction in the prevalence of older patients with DM treated with ACE-I/ARBs, from 87.3% in 2010 to 78.3% in 2022, in the polypharmacy group. A reduction in the prevalence of older DM patients treated with diuretics was observed in both groups overtime. In general, both groups experienced a reduction in exposure to Ca-antagonists, antiplatelet drugs, while there was an increase in the use of anticoagulants, beta-blockers, and lipid-lowering drugs.

**Table 1:** Baseline characteristics of older patients with DM by polypharmacy exposure in 2010, 2016 and 2022

	2010 *N* = 243,160		2016 *N* = 284,350		2022 *N* = 314,238	
	Polypharmacy		Polypharmacy		Polypharmacy	
	Yes *N* = 33,565	No *N* = 209,595	Yes *N* = 40,768	No *N* = 243,582	Yes *N* = 36,991	No *N* = 277,247
Age (ys), mean ± SD	76.5 ± 6.4	74.7 ± 6.3	77.4 ± 6.5	75.2 ± 6.5	78.0 ± 6.5	75.8 ± 6.6
Gender, *N* (%)						
Female	16,086 (47.9%)	104,829 (50.0%)	18,761 (46.0%)	114,960 (45.4%)	15,968 (43.2%)	124,768 (45.0%)
ATS, *N* (%)						
Città Metropolitana Di Milano	10,855 (32.3%)	72,064 (34.4%)	12,950 (31.8%)	81,634 (33.5%)	11,606 (31.4%)	91,361 (33.0%)
Insubria	4250 (12.7%)	29,937 (14.3%)	5233 (12.8%)	35,600 (14.6%)	4970 (13.4%)	42,368 (15.3%)
Montagna	1037 (3.1%)	6590 (3.1%)	1307 (3.2%)	8223 (3.4%)	1127 (3.1%)	8146 (2.9%)
Brianza	3671 (10.9%)	24,531 (11.7%)	4364 (10.7%)	29,282 (12.0%)	3968 (10.7%)	34,212 (12.3%)
Bergamo	3532 (10.5%)	21,627 (10.3%)	4557 (11.2%)	25,450 (10.5%)	3976 (10.8%)	29,562 (10.7%)
Brescia	4763 (14.2%)	23,860 (11.4%)	5302 (13.0%)	28,443 (11.7%)	4831 (13.1%)	32,537 (11.7%)
Val Padana	3150 (9.4%)	17,096 (8.2%)	3892 (9.6%)	19,110 (7.9%)	3451 (9.3%)	21,523 (7.8%)
Pavia	2178 (6.5%)	12,818 (6.1%)	2951 (7.2%)	14,216 (5.8%)	2855 (7.7%)	15,912 (5.7%)
Pre-existing conditions, *N* (%)						
Cerebrovascular disease	7385 (22.0%)	18,243 (8.7%)	7164 (17.6%)	17,691 (7.3%)	4849 (13.1%)	16,258 (5.9%)
Ischemic heart disease	16,128 (48.1%)	28,996 (13.8%)	16,601 (40.7%)	31,544 (13.0%)	13,086 (35.4%)	35,205 (12.7%)
Acute and chronic heart failure	8744 (26.1%)	10,803 (5.2%)	9724 (23.9%)	11,306 (4.6%)	8085 (21.9%)	13,005 (4.7%)
Atrial fibrillation	6530 (19.5%)	12,370 (5.9%)	7527 (18.5%)	12,704 (5.2%)	6666 (18.0%)	13,878 (5.0%)
Peripheral artery disease	4573 (13.6%)	8525 (4.1%)	4146 (10.2%)	7708 (3.2%)	2452 (6.6%)	6183 (2.2%)
Kidney disease	4598 (13.7%)	5154 (2.5%)	5389 (13.2%)	5940 (2.4%)	4027 (10.9%)	6169 (2.2%)
Liver disease	3112 (9.3%)	12,233 (5.8%)	2186 (5.4%)	7621 (3.1%)	1199 (3.2%)	4253 (1.5%)
Gastrointestinal disorders	3388 (10.1%)	9211 (4.4%)	2763 (6.8%)	6788 (2.8%)	1602 (4.3%)	5109 (1.8%)
Chronic obstructive pulmonary disease or Respiratory failure	6701 (20.0%)	11,940 (5.7%)	6734 (16.5%)	9981 (4.1%)	5793 (15.7%)	13,013 (4.7%)
Cancer	7437 (22.2%)	35,398 (16.9%)	8844 (21.7%)	39,358 (16.2%)	7516 (20.3%)	39,878 (14.4%)
At least one comorbidity*	27,409 (81.7%)	91,358 (43.6%)	31,042 (76.1%)	96,136 (39.5%)	19,777 (72.6%)	101,748 (37.5%)
Medications, *N* (%)						
ACE-I or ARBS	29,291 (87.3%)	142,182 (67.8%)	33,826 (83.0%)	169,593 (70.3%)	28,961 (78.3%)	186,583 (67.3%)
Beta blockers	18,816 (56.1%)	58,587 (28.0%)	26,686 (65.5%)	87,591 (36.3%)	26,195 (70.8%)	113,62 (41.0%)
Diuretics	20,246 (60.3%)	40,963 (19.5%)	24,160 (59.3%)	46,114 (19.1%)	20,203 (54.6%)	47,792 (17.2%)
Ca-antagonists	18,891 (56.3%)	64,286 (30.7%)	19,482 (47.8%)	66,999 (27.8%)	15,948 (43.1%)	66,345 (23.9%)
Lipid lowering drugs	23,918 (71.3%)	95,656 (45.6%)	31,552 (77.4%)	137,141 (56.9%)	30,146 (81.5%)	174,27 (62.9%)
Antiplatelet drugs	25,354 (75.5%)	89,669 (42.8%)	27,878 (68.4%)	76,425 (31.7%)	22,584 (61.1%)	84,911 (30.6%)
Oral anticoagulant drugs	5616 (16.7%)	12,353 (5.9%)	8519 (20.9%)	17,295 (7.2%)	10,294 (27.8%)	26,280 (9.5%)
Respiratory drugs	9090 (27.1%)	24,393 (11.6%)	12,013 (29.5%)	32,576 (13.5%)	7592 (20.5%)	23,608 (8.5%)

Kidney disease includes chronic kidney disease or end stage; respiratory drugs include chronic obstructive pulmonary disease or asthma drugs

Abbreviations: *SD* standard deviation, *ATS* Agenzie di tutela della salute (Health protection agencies), *ACE-I* Angiotensin-converting-enzyme inhibitors, *ARBS* Angiotensin II receptor blockers

*At least one comorbidity: among the pre-existing conditions reported in Table 1

**Fig. 2:**
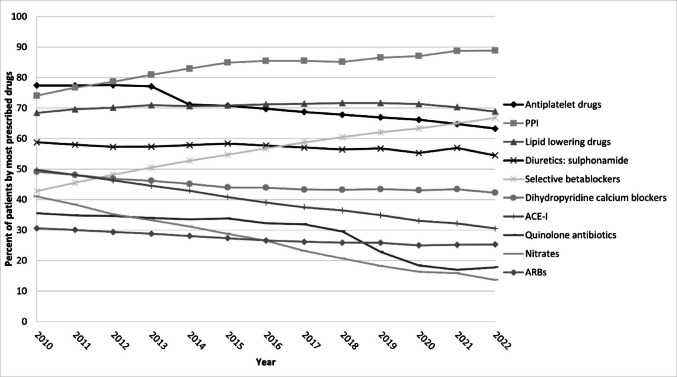
Trend of most prescribed drug classes in older patients with diabetes in polypharmacy. *ACE-I* angiotensin-converting enzyme inhibitors, *ARBs* angiotensin II receptor agonist blockers, *PPI* proton pump inhibitors. In the figure are plotted the percentage of subjects with at least one prescription of a drug at the 4th ATC classification level by index year

**Fig. 3:**
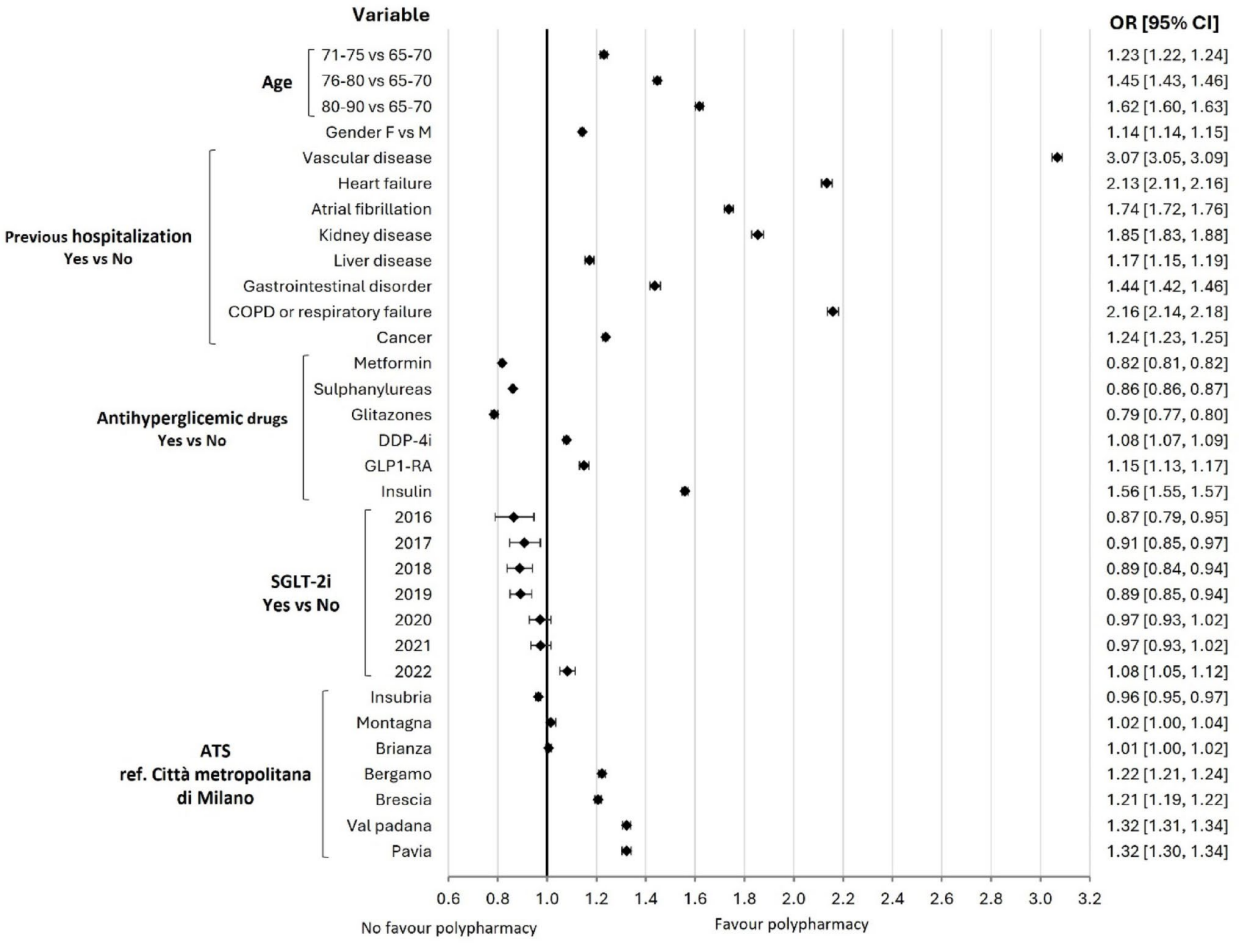
Adjusted Odds ratio (95% CI) in older patients with diabetes of being exposed to polypharmacy. *F* female, *M* male, *DPP-4i* dipeptidyl peptidase-4 inhibitors, *GLP-1 RA* Glucagon-like peptide-1 receptor agonists, *COPD* chronic obstructive pulmonary disease, *SGLT-2i* sodium-glucose co-transporter-2 (SGLT2) inhibitors, * ATS* Agenzia di Tutela della Salute (Health Protection Agencies)

Overall, the prevalence of older patients with DM exposed to polypharmacy increased from 13.8% in 2010 to 15.8% in 2013. However, a decrease was observed over time, with a notable drop in 2020 (11.8%) and 2021 (9.1%), followed by an increase to 11.7% in 2022 (Fig. 1, Table 1S). The trend was similar when stratified by age group, (Fig. 1, Table 1S) sex, with a higher prevalence in older patients (76–80 and 81–90 years) and males (Fig. 2S, Table 1S). Although differences in the prevalence of patients in polypharmacy were seen by ATS, a similar trend was observed over time (Fig. 3S).

Table 2 displays the frequency and percentage of prescriptions for antihyperglycemic drug classes according to polypharmacy exposure in 2010, 2016, and 2022. Older patients with DM exposed to polypharmacy were less frequently treated with metformin and sulfonylureas, but more frequently treated with insulin compared to those not exposed. Similar findings were observed in 2016 and 2022; however, a lower percentage of older patients were prescribed sulfonylureas in 2022 compared to 2010 in both groups. The percentage of patients treated with DPP-4i was similar in both groups, with a notable increase in the use since 2016.

**Table 2:** Frequencies and percentage of older patients with diabetes by antihyperglycemic drugs and polypharmacy

	2010		2016		2022	
	Polypharmacy		Polypharmacy		Polypharmacy	
	Yes *N* = 33,565	No *N* = 209,595	Yes *N* = 40,768	No *N* = 243,582	Yes *N* = 36,991	No *N* = 277,247
Metformin	19,237 (57.3%)	148,416 (70.8%)	22,759 (55.8%)	181,757 (74.6%)	22,248 (60.1%)	219,504 (79.2%)
Sulphonylureas	14,537 (43.3%)	116,404 (55.5%)	10,921 (26.8%)	85,459 (35.1%)	5295 (14.3%)	49,660 (17.9%)
Glinides	5161 (15.4%)	20,053 (9.6%)	5147 (12.6%)	19,111 (7.9%)	1321 (3.6%)	7234 (2.6%)
Glitazones	1050 (3.1%)	13,012 (6.2%)	1554 (3.8%)	18,194 (7.5%)	1530 (4.1%)	20,380 (7.4%)
Acarbose	944 (2.8%)	4713 (2.3%)	2029 (5.0%)	10,118 (4.2%)	1272 (3.4%)	7281 (2.6%)
DPP4i	520 (1.6%)	4369 (2.1%)	5709 (14.0%)	34,026 (14.0%)	7824 (21.2%)	53,452 (19.3%)
Insulin	12,098 (36.0%)	35,246 (16.8%)	16,287 (40.0%)	49,635 (20.4%)	13,364 (36.1%)	55,650 (20.1%)
GLP-1 RA	139 (0.4%)	913 (0.4%)	714 (1.8%)	4827 (2.0%)	5741 (15.5%)	41,103 (14.8%)
SGLT-2i	–	–	608 (1.5%)	4511 (1.9%)	8003 (21.6%)	47,962 (17.3%)

Abbreviation: *DPP-4i* dipeptidyl peptidase-4 inhibitors, *GLP-1 RA* Glucagon-like peptide-1 receptor agonists, *SGLT-2i* Sodium-glucose co-transporter-2 (SGLT2) inhibitors

In 2022, when prescriptions for GLP-1RA and SGLT-2i became more prevalent, we observed a higher percentage of older patients with DM exposed to polypharmacy receiving SGLT-2i (21.6%) compared to those not exposed (17.3%), while no difference was seen in GLP-1RA prescriptions between the two groups (Table 2). When stratifying the population by hospitalizations for HF and vascular diseases, we still observed a higher prevalence of patients prescribed SGLT-2i than those receiving GLP-1RA in both groups (Table 2S).

Figure 2 illustrates the trend of use of the ten most prescribed drugs at the 4th level of the ATC classification, from 2010 to 2022, in the polypharmacy group. Proton-pump inhibitors (PPIs) were the most prescribed drugs, increasing by nearly 15% from 2010 to 2022. There was also a 20% rise in the use of selective beta-blockers, mainly due to an increase in bisoprolol usage, from 37.0% in 2010 to 64.5% in 2022 (Fig. 4S). Conversely, a decline in the prevalence of ACE-I was observed, from 49.7% in 2010 to 30.5% in 2022 (Fig. 2).

ORs estimates and their corresponding 95% CIs for the probability of being exposed to polypharmacy are presented in Fig. 3. Older patients with DM were more likely to be exposed to polypharmacy than younger patients, females more than males, and patients with comorbidities. The use of DPP-4i, GLP-1RA, and insulin was significantly associated with a higher probability of polytherapy. The effect of SGLT-2i varied by year, with an opposite OR favouring the polytherapy group in 2022 compared to 2016. An effect in favor of polypharmacy in the areas of ATS Bergamo, ATS Brescia, ATS Val Padana, and ATS Pavia compared to ATS Città Metropolitana of Milan was observed.

## Discussion

This study evaluated the trends in chronic polypharmacy and antihyperglycemic medications within a relatively large cohort of elderly patients with DM. Our findings indicated an increase in the number of elderly DM patients from 2010 to 2022. Notably, there was a decrease in the prevalence of polypharmacy exposure, despite an initial increase up to 2013. The significant reduction in polypharmacy observed from 2014 may be partly attributed to the implementation of the deprescribing model, which involves the withdrawal of inappropriate or non-beneficial medications under the supervision of a healthcare professional. This approach aims to manage polypharmacy and improve both the quality of care and the quality of life [30, 31]. Furthermore, the notable decline in prescribing patterns observed in 2020 and 2021 may have been influenced by the COVID-19 pandemic, reduced access to outpatient visits during the pandemic years, and the consequent difficulty in managing access to therapies, rather than by a change in physicians’ prescribing habits in patients on polypharmacy [32]. Indeed, several studies have highlighted how the COVID-19 pandemic impacted standard clinical care, including primary care for people with T2D, visits for cardiovascular events, and cancer diagnoses [33–35]. This trend is consistent with a previous study reporting a decrease in prescriptions of antihyperglycemic and antihypertensive drugs among elderly Canadian patients, followed by a subsequent increase after the pandemic [33].

Additionally, we evaluated the predictors associated with exposure to polypharmacy in this population and found that older age, female sex, the presence of comorbidities, and the use of DPP-4 inhibitors, GLP-1 RA, and insulin were associated with a higher probability of being exposed to polypharmacy. The use of SGLT-2i in 2022 was also associated with a higher risk of polypharmacy, similar to that of GLP-1 RAs. SGLT-2i, being newer than GLP-1 RAs, likely required a learning period for physicians. The use of these newer drug classes with cardiovascular and renal protective effects likely reflects the coexistence of cardiovascular and/or renal complications. Indeed, elderly patients exposed to polypharmacy had more comorbidities, including cerebrovascular and ischemic heart disease, HF, AF, and chronic obstructive pulmonary disease or respiratory failure, compared to those not exposed. These findings align with those of a recent study conducted in a Danish population with T2D (aged ≥ 18 years), which reported an increase in polypharmacy from 53% in 2000 to 76% in 2020, particularly among patients with greater multimorbidity [36]. However, while the Danish study showed a steady rise, our results demonstrated an inverse trend since 2015.

We also had the opportunity to analyze changes in the prescription of antihyperglycemic drugs over time. Older patients with DM exposed to polypharmacy were less frequently treated with metformin and sulfonylureas but were more likely to receive insulin compared to those not exposed. Notably, we observed a significant decline in sulfonylurea use in both groups since 2016, with the most pronounced decrease occurring in 2022. Additionally, the use of GLP-1 RA and DPP-4 inhibitors was similar in both groups, with a substantial increase in use since 2022 and 2016, respectively. In 2022, a higher percentage of patients exposed to polypharmacy were treated with SGLT-2i (21.6%) compared to those not exposed to polypharmacy (17.3%).

The increased use of drugs with proven CV protection in older DM patients, along with the reduction in prescriptions of sulfonylureas (as this drug class is generally advised with caution in the elderly), reflects adherence to recommended GLs [3, 27, 28, 37, 38]. Furthermore, DPP-4 inhibitors are recommended for older individuals with T2D due to their CV safety, negligible risk of hypoglycemia, and minimal adverse effects [3, 27, 28, 38–40].

In our analysis, PPIs were the most prescribed drug class for older patients with DM, showing an increase of nearly 15% from 2010 to 2022. This trend aligns with similar observations in the general population with T2D [36]. The high use of PPIs could be due to the concomitant use of antiplatelet therapy in elderly people, which, although decreasing over the years, remains high. Indeed, the prescription of PPIs is reimbursed by the NHS for the prevention of upper gastrointestinal tract complications in patients receiving chronic treatment with antiplatelet and nonsteroidal anti-inflammatory drugs in advanced age, as well as in those undergoing concomitant therapy with anticoagulants or corticosteroids. Furthermore, we observed an increase in the use of oral anticoagulants, which, particularly in the polypharmacy group, reflects the higher prevalence of AF and aligns with international GL recommendations [8, 12–24]. Additionally, there was a 20% increase in the use of selective beta-blockers, primarily due to the rising use of bisoprolol, which increased from 37.0% in 2010 to 64.5% in 2022. Conversely, we observed a 9% reduction in the prescription of ACE-I and ARBs in the polypharmacy group from 2010 to 2022, largely driven by a 19% decrease in the use of ACE-I.

The markedly increase in the use of selective beta-blockers observed is consistent with a previous study showing a high prescription rate of beta-blockers, particularly bisoprolol, in older patients with AF, especially in the subgroup with T2D [37]. In our study, older patients with DM exposed to polypharmacy had higher rates of HF and AF compared to those not exposed. Beta-blockers are known to reduce mortality and HF hospitalization and improve functional status in patients with HF with reduced ejection fraction (HFrEF), with or without diabetes [41–43]. The treatment benefits strongly support using beta-blockers in patients with HFrEF and diabetes [43]. Moreover, beta-blockers are recommended as first-choice drugs in patients with AF to control heart rate and reduce symptoms [44].

However, Italian regulatory agencies suggest caution in the use of beta-blockers, such as carvedilol and metoprolol, in patients with DM due to the risk of worsening blood glucose control or masking the symptoms of hypoglycemia. Furthermore, metoprolol should be administered with caution in the elderly due to the increased likelihood of adverse events. Conversely, bisoprolol has been shown to be effective in reducing all-cause mortality and hospitalization for HF, even in elderly patients and those with DM or impaired renal function [45].

The trends observed suggest that clinicians’ approaches to treating older patients with DM are increasingly guided by clinical conditions and aligned with appropriate, guideline-based medication use over time. Findings from previous studies indicate that inappropriate medication use is less common among individuals with T2D exposed to polypharmacy compared to the general population. This is likely due to the improvements in CV clinical outcomes observed with the use of recommended drugs [5–12, 36]. Consequently, it has been suggested that the absence of polypharmacy in T2D may reflect missed opportunities for risk reduction and prevention of multimorbidity. On the other hand, inappropriate prescriptions in older individuals with T2D have been linked to an increased risk of mortality compared to those appropriately treated [4]. This has led to the proposal that polypharmacy should be redefined based on guideline-driven appropriateness rather than merely the number of medications prescribed [12].

The present study has several strengths and limitations that should be considered. A major strength is that this analysis was conducted in a large, unselected cohort of older individuals with DM receiving routine clinical care over the past decade. Furthermore, our findings are not influenced by the economic status of the individuals, as all Italian citizens have free access to the public healthcare system.

However, there are several limitations inherent to studies using administrative databases. For instance, data on drugs not reimbursed by the NHS, such as benzodiazepines, are not available, which may lead to an underestimation of polypharmacy. Additionally, clinical variables (e.g., renal function, BMI, glycemic levels) are not reported in our database, preventing us from accounting for these confounders in our analysis. We could not distinguish between type 1 and type 2 diabetes due to missing information in our database; however, more than 90% of patients with DM are estimated to have type 2 [46]. Another limitation is the potential for bias by indication. Although we adjusted for multiple confounders, the risk of residual confounding remains.

In conclusion, our analysis showed an increase in the proportion of older patients with DM exposed to polypharmacy in the early years of the observation period. However, since 2014, an opposite trend has been observed, continuing until 2021. Furthermore, the observed changes in the prescribing patterns of recommended drugs (including glucose-lowering agents and medications for CV complications) may reflect a shift in clinicians’ attitudes towards better management of this population. This shift is likely based not only on the clinical profiles of the patients but also on alignment with recent evidence-based data. Therefore, rather than focusing solely on the numerical definition of polypharmacy, it would be more meaningful to consider the appropriateness of medication use and adherence to guidelines in older patients with DM, especially those with comorbidities, as has been recently suggested [12]. Polypharmacy is an important issue not only from a public health perspective but also in terms of increasing healthcare costs.

## Electronic supplementary material

Below is the link to the electronic supplementary material.

Supplementary Material 1
