# Supplementary material for: Polypharmacy in older patients with diabetes mellitus: a population based-study of northern Italy
Source: Acta Diabetol. 2025 Jun 5;62(11):1929–39. doi: 10.1007/s00592-025-02523-1 (PMC12640330; doi:10.1007/s00592-025-02523-1)
Supplement: Supplementary file 1 — Supplementary Material 1 [file 592_2025_2523_MOESM1_ESM.docx]

**SUPPLEMENTARY MATERIAL**

**Polypharmacy in Older Patients with Diabetes Mellitus: A Population Based-Study of Northern Italy**

Figure 1S

Table 1S

Figure 2S

Table 2S

Figure 3S

Figure 4S

Appendix

**Figure 1S.** Study flowchart


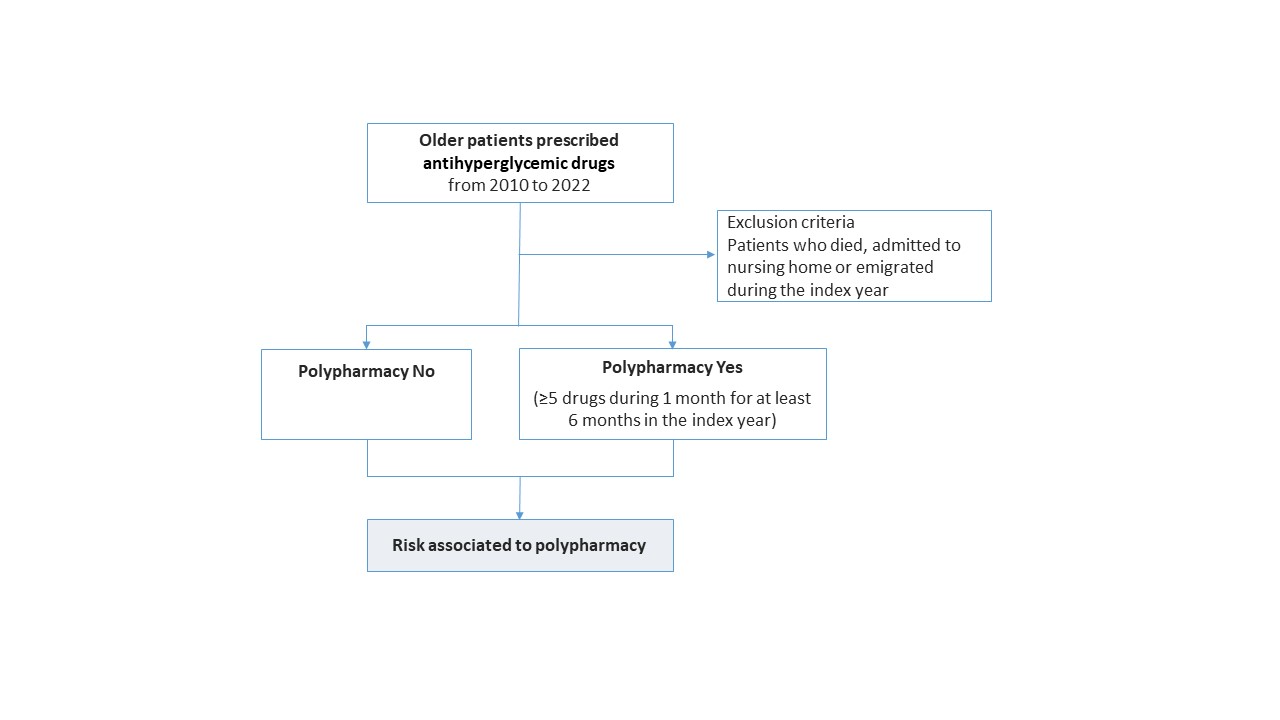


**Tab 1S**. Trend of polypharmacy in older patients with diabetes, overall and stratified by age and gender.

|  | | **Year** | | | | | | | | | | | | |
| --- | --- | --- | --- | --- | --- | --- | --- | --- | --- | --- | --- | --- | --- | --- |
|  | | **2010**  **(N/D, %)** | | | **2011**  **(N/D, %)** | | | **2012**  **(N/D, %)** | | | **2013**  **(N/D, %)** | | **2014**  **(N/D, %)** | |
| **All subjects** | | 33,565 / 243,160  (13.8%) | | | 36,788 / 253,197  (14.5%) | | | 40,837 / 262,848  (15.5%) | | | 42,907 / 271,428  (15.8%) | | 42,937 / 277,605  (15.5%) | |
| **Gender** | **Female** | 16,086 / 120,915  (13.3%) | | | 17,431 / 124,862  (14.0%) | | | 18,909 / 128,316  (14.7%) | | | 19,786 / 131,183  (15.1%) | | 19,875 / 132,956  (15.0%) | |
|  | **Male** | 17,479 / 122,245  (14.3%) | | | 19,357 / 128,335  (15.1%) | | | 21,928 / 134,532  (16.3%) | | | 23,121 / 140,245  (16.5%) | | 23,056 / 144,649  (15.9%) | |
| **Age class**  **(years)** | **65-70** | 6,951 / 70,212  (9.9%) | | | 7,361 / 71,532  (10.3%) | | | 7,927 / 73,512  (10.8%) | | | 8,375 / 75,622  (11.1%) | | 8,131 / 76,884  (10.6%) | |
|  | **71-75** | 8,465 / 66,367  (12.8%) | | | 9,128 / 69,012  (13.2%) | | | 10,120 / 71,681  (14.1%) | | | 10,272 / 72,685  (14.1%) | | 9,673 / 71,369  (13.6%) | |
|  | **76-80** | 8,651 / 54,773  (15.8%) | | | 9,403 / 57,066  (16.5%) | | | 10,400 / 58,919  (17.7%) | | | 11,033 / 61,255  (18.0%) | | 11,288 / 64,466  (17.5%) | |
|  | **81-90** | 9,498 / 51,808  (18.3%) | | | 10,896 / 55,587  (19.6%) | | | 12,390 / 58,736  (21.1%) | | | 13,227 / 61,866  (21.4%) | | 13,839 / 64,886  (21.3%) | |
|  | | **2015**  **(N/D, %)** | | **2016**  **(N/D, %)** | | | **2017**  **(N/D, %)** | | | **2018**  **(N/D, %)** | | **2019**  **(N/D, %)** | |  |
| **All subjects** | | 39,641 / 280,742  (14.1%) | | 40,768 / 284,350  (14.3%) | | | 39,611 / 284,176  (13.9%) | | | 38,885 / 288,776  (13.5%) | | 38,710 / 294,120  (13.2%) | |  |
| **Gender** | **Female** | 18,303 / 133,234  (13.7%) | | 18,761 / 133,721  (14.0%) | | | 17,980 / 132,160  (13.6%) | | | 17,327 / 134,580  (12.9%) | | 17,488 / 133,254  (13.1%) | |  |
|  | **Male** | 21,338 / 147,508  (14.5%) | | 22,007 / 150,629  (14.6%) | | | 21,631 / 152,016  (14.2%) | | | 21,383 / 159,574  (13.4%) | | 21,397 / 155,522  (13.8%) | |  |
| **Age class (years)** | **65-70** | 7,314 / 77,744  (9.4%) | | 7,544 / 79,231  (9.5%) | | | 6,948 / 76,313  (9.1%) | | | 6,202 / 73,242  (8.5%) | | 9,496 / 74,906  (8.7%) | |  |
|  | **71-75** | 8,597 / 69,174  (12.4%) | | 8,365 / 66,007  (12.7%) | | | 8,097 / 67,011  (12.1%) | | | 8,063 / 72,164  (11.2%) | | 8,216 / 69,056  (11.9%) | |  |
|  | **76-80** | 10,441 / 66,592  (15.7%) | | 10,865 / 68,859  (15.8%) | | | 10,693 / 69,505  (15.4%) | | | 10,021 / 69,570  (14.4%) | | 10,281 / 70,332  (14.6%) | |  |
|  | **81-90** | 13,289 / 67,232  (19.8%) | | 13,994 / 70,253  (19.9%) | | | 13,873 / 71,347  (19.4%) | | | 14,424 / 79,140  (18.2%) | | 13,892 / 74,482  (18.7%) | |  |
|  | | **2020**  **(N/D, %)** | **2021**  **(N/D, %)** | | | **2022**  **(N/D, %)** | | |  | | |  | |  |
| **All subjects** | | 34,217 / 289,628  (11.8%) | 27,256 / 298,485  (9.1%) | | | 36,991 / 314,238  (11.8%) | | |  | | |  | |  |
| **Gender** | **Female** | 15,048 / 131,895  (11.4%) | 11,984 / 135,093  (8.9%) | | | 15,968 / 140,736  (11.4%) | | |  | | |  | |  |
|  | **Male** | 19,169 / 157,733  (12.2%) | 15,272 / 163,392  (9.4%) | | | 21,023 / 173,502  (12.1%) | | |  | | |  | |  |
| **Age class (years)** | **65-70** | 5,318 / 70,960  (7.5%) | 4,170 / 72,237  (5.8%) | | | 5,741 / 75,787  (7.6%) | | |  | | |  | |  |
|  | **71-75** | 7,333 / 72,340  (10.1%) | 5,995 / 76,959  (7.8%) | | | 7,894 / 78,269  (10.1%) | | |  | | |  | |  |
|  | **76-80** | 8,639 / 66,283  (13.0%) | 6,512 / 64,941  (10.0%) | | | 8,961 / 70,078  (12.8%) | | |  | | |  | |  |
|  | **81-90** | 12,927 / 80,045  (16.2%) | 10,579 / 84,348  (12.5%) | | | 14,395 / 90,104  (16.0%) | | |  | | |  | |  |
| N: number of older patients with diabetes in polypharmacy; D: number of older patients with diabetes | | | | | | | | | | | | | |  |


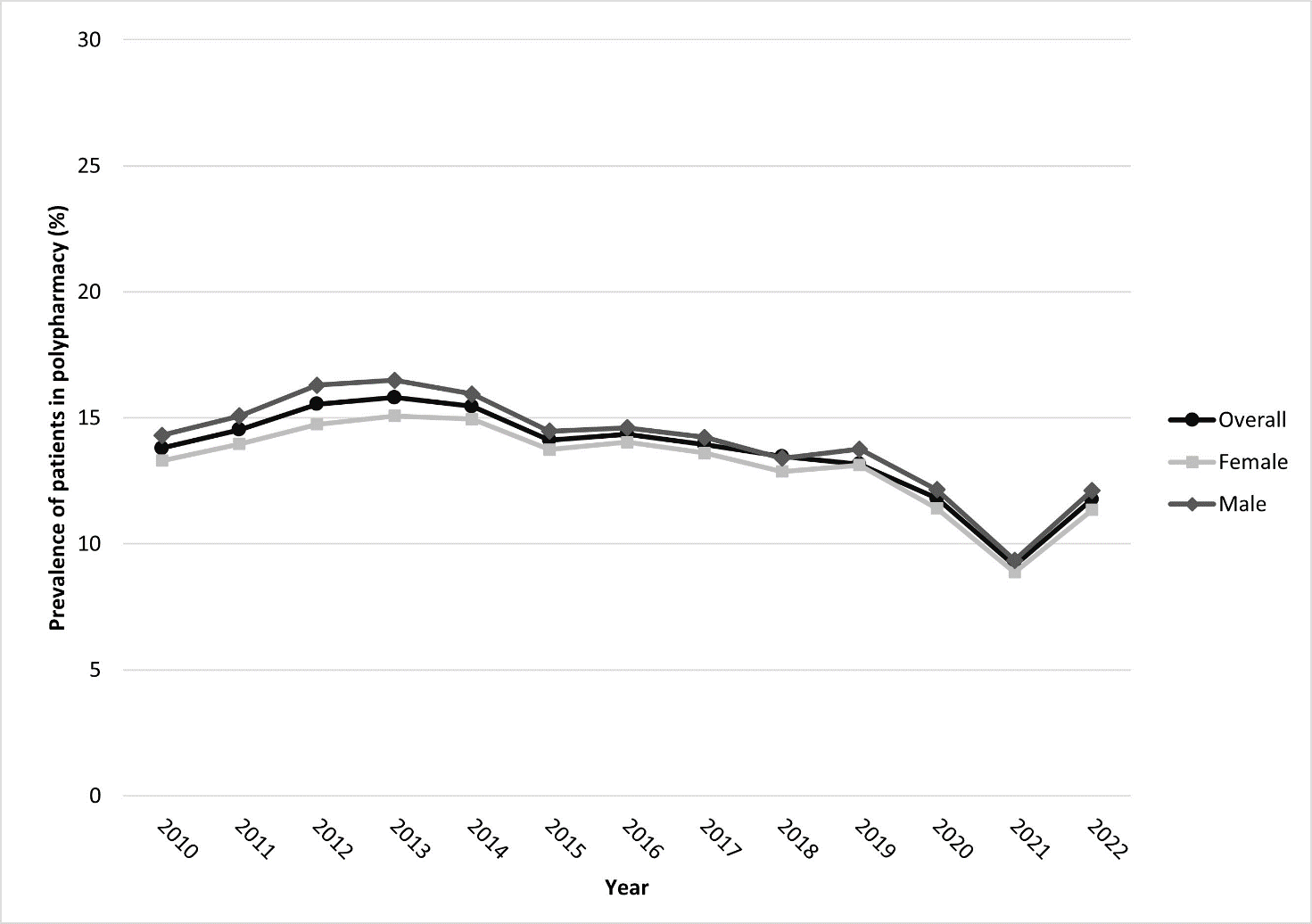
**Figure 2S.** Trend of polypharmacy in older patients with diabetes, overall and stratified by gender.

**
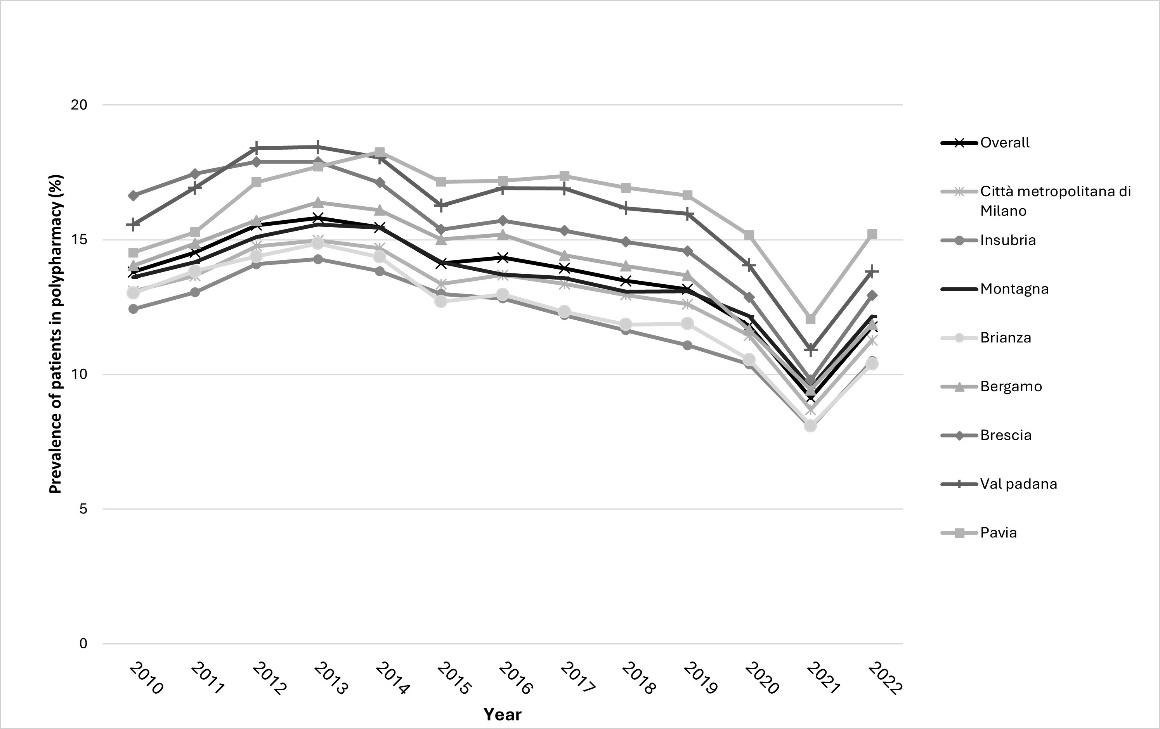
Figure 3S**. Trend of polypharmacy in older patients with diabetes, overall and according to ATS

*ATS: Agenzia di Tutela della Salute (Health Protection Agencies).*

**Table 2S.** GLP-1 and SGLT-2i prescriptions according to the occurrence of previous hospitalisations for heart failure and/or vascular disease in 2022

|  | **Year 2022** | | |
| --- | --- | --- | --- |
|  | **Previous HF** | **Previous vascular disease hospitalisation** | **Previous HF and vascular disease hospitalisation** |
|  | **N=9062** | **N=53,362** | **N=12,028** |
| **GLP-1 RA only** | 846  (9.34%) | 7205  (13.50%) | 1079  (8.97%) |
| **SGLT-2i only** | 2090  (23.06%) | 9973  (18.69%) | 3498  (29.08%) |
| **Abbreviation:** GLP-1 RA, Glucagon-like peptide-1 receptor agonists; SGLT-2i, Sodium-glucose co-transporter-2 (SGLT2) inhibitors. | | | |

**Figure 4S.** Prevalence of older patients with diabetes receiving betablockers by principle active

**
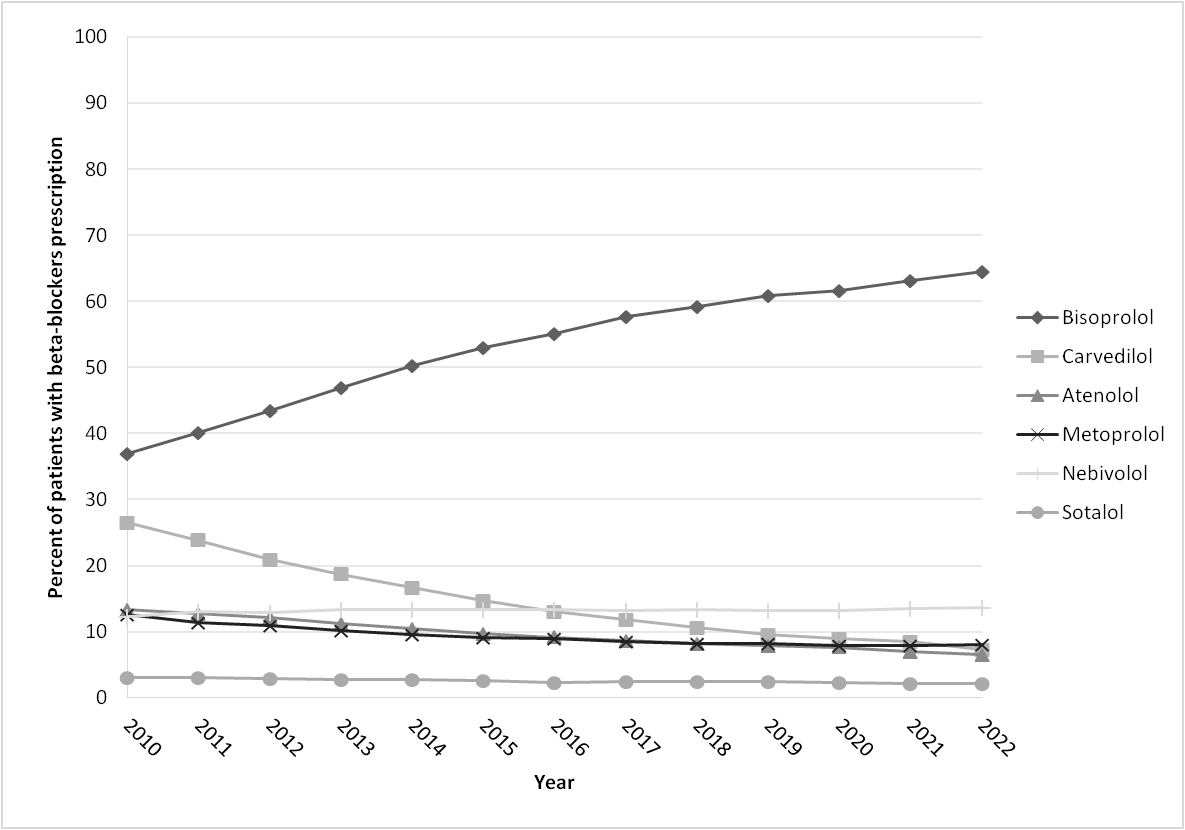
**

**APPENDIX**

**Anatomical Therapeutic Chemical Classification (ATC codes)**

| **Drugs** | **ATC-Code** |
| --- | --- |
| **Antihyperglycemic drugs** | |
| **Insulin** | A10A* |
| **Metformin** | A10BA*; A10BD01; A10BD02; A10BD03; A10BD05; A10BD07; A10BD08; A10BD10; A10BD11; A10BD13; A10BD14; A10BD15; A10BD16; A10BD17; A10BD18; A10BD20 |
| **Sulphonylureas** | A10BB*; A10BD01; A10BD02; A10BD04; A10BD06 |
| **Glinides** | A10BX02; A10BX03; A10BX08; A10BD14 |
| **Acarbose** | A10BF*; A10BD17 |
| **Glitazones** | A10BG*; A10BD03; A10BD04; A10BD05; A10BD06; A10BD09; A10BD12 |
| **DPP-4i** | A10BH*; A10BD07; A10BD08; A10BD09; A10BD10; A10BD11; A10BD12; A10BD13; A10BD18; A10BD19; A10BD21; |
| **GLP1-RA** | A10BJ* |
| **SGLT-2i** | A10BK*; A10BX09; A10BX11; A10BX12; A10BD15; A10BD16; A10BD19; A10BD20; A10BD21 |
| **Other drugs** | |
| **ACE-I/ARBS** | C09*; C10BX10 |
| **Diuretics** | C03* |
| **Beta blockers** | C07* |
| **Ca-antagonists** | C08* |
| **Lipid-lowering drugs** | C10* |
| **Antiplatelet drugs** | N02BA01; B01AC*; C10BX08; C10BX02; C10BX05; C10BX01 |
| **Oral anticoagulants** | B01AA03; B01AA07; B01AE07; B01AF01; B01AF02; B01AF03 |
| **Respiratory drugs** | R03AC*, R03AK*, R03BA*, R03BB*, R03DC* |

**Abbreviations:** DPP-4i, dipeptidyl peptidase-4 inhibitors; GLP-1 RA, Glucagon-like peptide-1 receptor agonists; SGLT-2i, Sodium-glucose co-transporter-2 (SGLT2) inhibitors; ACE-I, angiotensin-converting enzyme inhibitors; ARBs, angiotensin II receptor agonist blockers

**International Classification of Disease, Ninth Revision DM (ICD9-CM code) for diagnosis and procedure**

| **Diagnosis and procedures** | **ICD-9 Code** |
| --- | --- |
| **Cerebrovascular disease** | 430, 431, 432.x, 433.xx, 434.xx, 435.x, 436 |
| **Ischemic heart disease** | 410.x, 411, 411.1, 411.8, 411.81, 411.89, 413, 413.0, 413.1, 413.9, 414.x, 36.x, 0066 |
| **Heart failure** | 398.91, 402.01, 402.11, 402.91, 404.01, 404.11, 404.91, 404.03, 404.13, 404.93, 428.xx |
| **Peripheral artery disease** | 440.2x, 440.3x, 443.81, 39.50, 38.18, 38.08, 39.90, 39.25, 39.26, 39.29 |
| **Atrial fibrillation** | 427.31, 427.32 |
| **Chronic kidney disease or end-stage kidney disease** | 585.x; V451, V560, V561, V562, V563.1, V563.2, V568, 38.95, 39.27, 39.42, 39.43, 39.95, 54.98, V420, 996.81, 55.61, 55.69 |
| **Lung disease**  (chronic obstructive disease and respiratory failure) | 491.x, 492.x, 494.x, 496.x, 518.81, 518.83, 518.84 |
| **Liver disease** | 070.x, 570.x, 571.x, 572.x, 573.x |
| **Gastrointestinal disorders** | Between 530 and 535 |
| **Cancer** | between 140 and 165, between 170 and 208, between 210 and 239, 2592, 28983 |
| **Vascular disease** (used for logistic regression) | 433.xx, 434.xx, 435.x, 436, 410.x, 411, 411.1, 411.8, 411.81, 411.89, 413, 413.0, 413.1, 413.9, 414.x, 36.x, 0066, 440.2x, 440.3x, 443.81, 39.50, 38.18, 38.08, 39.90, 39.25, 39.26, 39.29 |
